# Study of Tribological Characteristics on the Surface of Railway Transport Components Using Atmospheric Plasma

**DOI:** 10.3390/ma19112208

**Published:** 2026-05-24

**Authors:** Denys Baranovskyi, Maryna Bulakh, Sergey Myamlin, Nikolay Sergienko, Sergey S. Myamlin

**Affiliations:** 1 Faculty of Mechanics and Technology, Rzeszow University of Technology, Aleja Powstańców Warszawy 12, 35-329 Rzeszow, Poland; d.baranovsky@prz.edu.pl; 2 Department of Wagon Engineering and Product Quality, Ukrainian State University of Railway Transport, Feuerbakh Sq. 7, 61050 Kharkiv, Ukraine; sergeymyamlin@gmail.com (S.M.); sergeymyamlin91@gmail.com (S.S.M.); 3 Institute of Education and Science in Mechanical Engineering and Transport, National Technical University “Kharkiv Polytechnic Institute”, Kyrpychova St. 2, 61002 Kharkiv, Ukraine; nesergienko@gmail.com

**Keywords:** tribological characteristics, surface treatment, atmospheric plasma, roughness, railway transport components, wear

## Abstract

This paper presents a comprehensive investigation of the effects of atmospheric plasma treatment (APT) on the surface morphology, microhardness, chemical composition, and tribological performance of alloy steel used in railway applications. A novel mathematical model is proposed to describe the dependence of the maximum surface asperity height on the APT parameters and material properties. Experimental validation was performed using a series of alloy steel specimens treated under controlled APT conditions. The surfaces were characterized by roughness measurements, microhardness testing, scanning electron microscopy, and energy-dispersive spectroscopy. Tribological properties were evaluated under dry sliding conditions using ball-on-disk tests with steel counterbodies (grades 1.3529 and 1.3505). Tribological testing showed that APT leads to a 6–7% reduction in the steady-state friction coefficient, eliminates the long running-in stage, and improves stability by lowering the coefficient of variation by up to 43%. Overall, this study demonstrates that APT provides a dual benefit: improving tribological performance through surface smoothing and stabilization of the friction regime, and preserving the mechanical and chemical integrity of the material.

## 1. Introduction

### 1.1. Background

The railway industry plays a strategic role in modern transportation systems, ensuring the efficient, reliable, and sustainable mobility of goods and passengers. With the growing demand for higher train speeds, increased axle loads, and extended service intervals, the requirements for railway components have become significantly more stringent. Key parts such as wheels, axles, brake systems, couplings, and structural elements of rolling stock are subjected to complex combinations of mechanical stresses, thermal fluctuations, and corrosive environments. Failures in these components not only lead to costly maintenance but may also compromise operational safety [1,2].

In this context, alloy steels remain the dominant material for railway transport components due to their high strength-to-weight ratio, wear resistance, and ability to withstand impact and cyclic loading [2,3]. Nevertheless, the performance of alloy steels is strongly influenced by the state of their surface layers, which serve as the primary zone of contact during frictional interaction [4]. Parameters such as the surface roughness, hardness, residual stresses, and chemical composition largely determine the tribological behavior of these steels. Even minor deviations in surface quality can significantly alter the friction coefficients, accelerate wear, and trigger fatigue cracks, thereby reducing component lifespan [5,6].

Surface roughness, in particular, has a pronounced effect on the functional properties. High roughness leads to increased contact stresses, localized heating, and micro-welding of asperities, all of which intensify adhesive and abrasive wear [7,8]. Moreover, rough surfaces exhibit higher susceptibility to corrosion, as irregularities act as stress concentrators and sites for chemical attack. Conversely, smoother surfaces provide better load distribution, lower friction, and improved fatigue strength, resulting in enhanced operational stability and reduced energy consumption in mechanical systems [9,10].

Therefore, improving the surface condition of alloy steels is a critical engineering task for railway applications. Solutions must balance the dual objectives of reducing microroughness while preserving or even enhancing the bulk mechanical properties of the steel. This requires advanced technologies that are not only effective and precise but also energy-efficient and environmentally sustainable, considering the scale of production and the long service life expected from railway components [10,11].

### 1.2. Analysis of Previous Studies

A considerable body of research has been devoted to the improvement in surface quality in alloy steels, with conventional methods such as grinding, polishing, honing, and abrasive blasting being the most widely applied. These techniques are effective in reducing roughness to a certain extent, but they often face limitations in industrial practice. For example, grinding and polishing are labor-intensive, time-consuming, and unsuitable for large-scale or complex-shaped components. In addition, they may introduce undesirable surface defects such as residual stresses, microcracks, or thermal damage, which in turn reduce fatigue resistance [12,13].

Alternative methods based on thermal and chemical treatments, such as laser surface treatment, plasma arc processing, or chemical etching, have been explored to overcome these limitations. These approaches can modify the surface layer at the microstructural level, leading to improved hardness, wear resistance, and corrosion protection. However, they also have significant drawbacks. Laser treatment, for instance, may cause excessive heating and alteration in the base material, while plasma arc processing often requires controlled environments and high energy input. Chemical treatments, on the other hand, pose environmental and safety concerns due to the use of aggressive reagents [14].

Numerous studies have addressed the challenge of improving the surface quality and tribological behavior of steels using various plasma-based or hybrid finishing methods. For instance, Lyu et al. [15] demonstrated that laser-enhanced plasma polishing enables the smoothing of fused silica surfaces at atomic and near-atomic scales, which confirms the capacity of plasma-based processes to achieve extremely fine surface finishes. However, these studies were primarily limited to optical materials and did not consider the performance of structural steels under tribological loads. Similarly, Inoue and Furushima [16] proposed predictive models for microforming limit curves that accounted for surface roughening, underlining the strong influence of surface topography on mechanical formability, though their work was directed at microforming processes rather than sliding wear.

Other research has focused on the in-process monitoring of surface quality. Fischer and Stöbener [17] introduced a methodology for in-process roughness inspection during sheet rolling, which enables the real-time control of surface characteristics in production. Although effective, this approach remains diagnostic rather than corrective. Furthermore, Yamaguchi et al. [18] investigated the magnetic field-assisted finishing of additively manufactured components, reporting improvements in both surface roughness and residual stress. Their findings highlight that hybrid physical methods can significantly enhance surface integrity; however, such techniques require specialized setups and remain relatively costly.

With respect to steels, Deng et al. [12] explored the effects of plasma beam polishing on AISI 304 stainless steel, showing that optimized parameters could reduce roughness significantly. Similarly, Sharma and Kumar [16], as well as Das and Chakraborty [14], studied optimization strategies for plasma arc cutting, mainly targeting the reduction in dross formation and improving the surface finish of cut edges. These works underline the potential of plasma technologies for surface quality improvement, yet they primarily concern cutting operations rather than controlled surface modification for tribological enhancement.

Importantly, most of the existing literature focuses on vacuum or controlled-gas plasma systems, which require costly infrastructure and are less practical for the large-scale treatment of railway components. Research on APT is still at an early stage, and investigations on how it influences the tribological properties of alloy steels used in railway transport are almost absent. This gap in knowledge is especially critical, considering that railway components operate under high contact pressures, fluctuating temperatures, and severe wear conditions, where surface roughness and friction stability play a decisive role in reliability.

### 1.3. State-of-the-Art Research

In recent years, plasma-assisted technologies have attracted growing attention as advanced methods for improving the surface properties of metals and alloys. Unlike conventional mechanical finishing, which removes material through abrasive action, or thermal processes that often alter the bulk microstructure, plasma-based treatments offer a more controlled and localized interaction with the material surface. This enables the reduction in microroughness by selectively melting and re-solidifying asperity peaks, while leaving the underlying structure largely unaffected.

Several pioneering works have confirmed the efficiency of plasma technologies in this context. For example, Deng et al. [17] demonstrated that plasma beam polishing significantly reduces the roughness values on stainless steel surfaces, producing smoother contact interfaces. Similarly, Yamaguchi et al. [15] emphasized that hybrid plasma-assisted finishing techniques can reduce both the roughness and residual stresses, which directly translates into improved fatigue resistance. Furthermore, Fischer and Stöbener [14] showed that integrating roughness inspection with plasma-assisted processes could lead to improved process control and higher reproducibility in industrial applications.

From an environmental and economic perspective, atmospheric plasma offers important advantages. Unlike vacuum plasma systems, APT operates under normal atmospheric conditions, eliminating the need for costly chambers, vacuum pumps, or controlled atmospheres. This makes it significantly more energy-efficient, scalable, and eco-friendly, as it avoids aggressive chemicals or excessive energy input. Moreover, APT systems can be easily integrated into existing production lines, making them attractive for large-scale applications such as railway transport engineering.

Despite these advances, current research still shows significant gaps. The majority of plasma-related studies have been carried out on stainless steels, non-ferrous alloys, ceramics, and biomedical materials [12,15,17,18,19]. Very few works have systematically addressed the effect of APT on alloy steels for railway components, particularly in terms of tribological performance such as friction coefficient stabilization, wear rate reduction, and counterbody protection. The absence of such studies limits the practical adoption of APT in railway applications, where operational reliability critically depends on surface quality.

Therefore, the present research aims to fill this gap by providing a comprehensive experimental and theoretical assessment of the effects of APT on alloy steel surfaces used in railway transport. By correlating surface roughness reduction, microstructural stability, and tribological performance, this study advances the understanding of how atmospheric plasma can be effectively employed in real-world engineering conditions.

### 1.4. Objectives

The objective of this study is to investigate the influence of atmospheric plasma treatment on the tribological characteristics of alloy steel surfaces used in railway transport components. The research focuses on:

- Analyzing the effect of APT on surface roughness parameters;
- Evaluating changes in microhardness, microstructure, and chemical composition after treatment;
- Studying tribological performance under dry sliding conditions, including friction coefficient stability and wear behavior;
- Validating theoretical models by experimental results to establish optimal treatment parameters.

### 1.5. Scientific Novelty

This work introduces, for the first time, a mathematical model describing the dependence of the maximum surface asperity height on the APT parameters and material properties. The study also provides a comprehensive experimental evaluation of alloy steel surfaces before and after treatment, demonstrating that APT can reduce roughness by 35–37% without altering the microhardness or chemical composition. The novelty lies in confirming that APT achieves dual functionality: improving tribological performance through surface smoothing, and maintaining the fundamental mechanical and chemical integrity of the material, making the method industrially viable for railway applications.

### 1.6. Paper Organization

The remainder of the paper is structured as follows: Section 2 presents the theoretical background of surface microroughness reduction during APT. Section 3 describes the materials and experimental methods used and discusses the results, including surface roughness, microhardness, microstructure, chemical composition, and tribological performance after APT. Section 4 concludes the study, highlighting the main findings, practical implications, and directions for future research.

## 2. Materials and Methods

Chapter 2 presents the theoretical and experimental methodology used to investigate the influence of atmospheric plasma treatment on the surface morphology and tribological behavior of alloy steel. Firstly, the physical mechanisms responsible for microroughness reduction during plasma exposure are described, including localized heating, selective melting of asperities, capillary-driven material redistribution, and rapid solidification of the surface layer. Next, a theoretical model relating the maximum asperity height to the plasma treatment parameters and material properties is developed. Finally, the experimental procedures used for validation of the proposed model are presented, including the surface roughness measurements, microhardness analysis, microstructural characterization, chemical composition evaluation, and tribological testing under room-temperature and elevated-temperature conditions.

### 2.1. Process Physics of Microroughness Reduction During APT

#### 2.1.1. Plasma Energy Absorption—Higher Energy Input at Asperity Peaks

Local heat flux from the plasma is more intense at protruding asperities due to direct exposure to the jet, higher local heat transfer coefficients, and limited conduction pathways into the bulk. The absorbed flux can be approximated as

(1) $$q_{abs}\;\approx \;\alpha_{\varepsilon }\cdot q_{conv}+\alpha_{i}\cdot q_{ion}+\alpha_{r}\cdot q_{rad},$$

where the terms represent convective transfer, ion bombardment/recombination, and radiation, respectively; $\alpha_{*}$ are fractions depending on the peak geometry and steel properties.

Asperities with small radii of curvature heat up faster than valleys, as expressed by

(2) $${\Delta T}_{peak}\left(t\right)\sim \;\left(\frac{q_{abs}}{k}\right)\cdot \sqrt{\frac{t}{\pi \alpha }}.$$

Peaks (smaller “conductivity root” $l_{d}=\sqrt{\alpha t}$ relative to the radius of curvature) heat up faster than valleys. This explains why peaks reach melting temperature earlier.

#### 2.1.2. Selective Melting of Asperities—Localized Softening of Protruding Micropeaks

A peak melts when the supplied energy exceeds the energy required to raise the surface to Tm and to overcome the latent heat of fusion:

(3) $$q_{abs}\cdot \tau \;\geq \;\rho \cdot c_{p}\left(T_{m}-T_{0}\right)\delta_{m}\;+\;\rho \cdot L_{f}\cdot \delta_{m},$$

where $\delta_{m}\;$ is the molten depth. This condition is satisfied at the protrusions earlier than at the depressions, due to the larger $q_{abs}$ and smaller heat dissipation. This threshold is reached first at asperities due to higher absorption and reduced dissipation. As a result, thin molten films form selectively on peaks without affecting the valleys, provided the process remains within the optimal power–time window.

#### 2.1.3. Surface Tension-Driven Flow—Redistribution of Molten Material from Peaks to Valleys

Once a thin molten film is formed, capillary forces drive material from high-curvature regions (asperities) into adjacent valleys. The Laplace pressure is

(4) Δp=γ·κ,

where κ is the surface curvature and γ is the surface tension of the melt.

In addition, Marangoni convection contributes since

(5) $$\frac{d\gamma }{dT}<0,$$

promoting flow from hotter to cooler zones.

The thin-film evolution can be described by lubrication theory:

(6) $$\frac{\partial h}{\partial t}=\nabla \cdot \left[\frac{\gamma h^{3}}{3\eta }\nabla \left(\nabla^{2}h\right)\right]\;+\;terms\;for\;Marangoni\;stresses.$$

For micron-scale asperities, the characteristic leveling time $\tau_{cap}$ lies in the μs–ms range, compatible with APT exposure.

#### 2.1.4. Solidification and Leveling—Reduction in Asperity Height and Overall Microroughness

After plasma removal, rapid cooling of the molten film occurs due to heat conduction into the bulk. Solidification proceeds from the substrate upward, governed by the Stefan number

(7) $$Ste=\frac{c_{p}\left(T_{m}-T_{0}\right)}{L_{f}}.$$

The final surface profile is thus ‘frozen’ in a leveled state: peaks are reduced and valleys partially filled. This results in a measurable decrease in $R_{a}$, $R_{z}$, and the maximum asperity height. Over-treatment, however, leads to deep melting, waviness, or cracking; hence, the optimal window must satisfy

(8) $$\tau_{cap}\leq \tau_{melt}\ll \tau_{bulk}.$$

### 2.2. Process Physics of Asperity Smoothing Under Atmospheric Plasma Treatment

**Figure 1:**
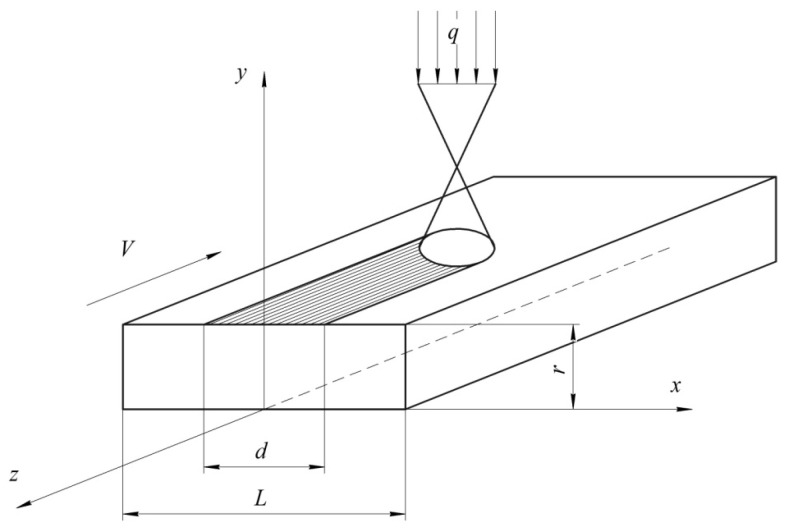
Schematic diagram of the thermal model of APT of the working surface of the sample material.

During the APT of the working surface of the material, a thermal process occurs that affects the change in the stress state of the surface being treated. For theoretical justification of the reduction in the roughness of the working surface of the material during APT, it is possible to use the possibility of considering the distributions of temperature and stresses of the surface layer of the material. For this purpose, the solution of the heat conductivity equation can be used. The thermal model can make it possible to estimate the parameters of APT, at which the effect of the melting of protrusions of surface microroughnesses can be obtained with or without the melting process. The thermal field in the zone of the APT of the working surface of the material can be reduced to the solution of the differential equation of heat conductivity. If the depth of the APT of the working surface of the material is less than the thickness of the sample or part, and has a gradual movement during the treatment, then the thermal field and the stress state can be calculated based on the proposed half-space model (Figure 1).

For this thermal model of the APT of the working surface of the sample material (Figure 1), the thermal physical coefficients of the material do not depend on temperature, and the radiation during APT is absorbed by the surface. In the quasi-stationary mode, the heat conductivity equation has the simplest form:

(9) $$\frac{\partial T}{\partial t}=\chi \nabla^{2}T,$$

with initial and limit conditions:

$$\frac{\partial T}{{\left.\partial Z\right|}_{z=0}}=-\frac{q_{0}K}{\lambda }exp\left(-kr^{2}\right);$$

(10) $$T\left(r,Z,0\right)=0;\;\underset{z\to \infty }{\lim}\;T\left(r,\;Z,\;t\right)<N;\;\underset{r\to \infty }{\lim}\;T\left(r,\;Z,\;t\right)<M,$$

where

χ,λ are the coefficients of thermal diffusivity and thermal conductivity;
r,Z are the values of the radius and the depth of action during APT;
K is the absorption coefficient;
∇ is the Hamilton operator;
k is the proportionality coefficient for the width of APT;
d is the width of APT;
T is the melting temperature of the material;
$q_{0}$ is the power of the APT of the material surface;
L is the width of the cross-section of the sample or part.

For the initial and limiting conditions for expression (9), we perform the Henkel transform in r and the Laplace transform in t. As a result, we obtain the following solutions:

(11) $$\begin{array}{c} T\left(r,\;Z,\;t\right)=\frac{q_{0}K}{4\lambda k}\int_{0}^{\infty }\left[J_{0}\left(\xi ,\;r\right)\cdot exp\left(-\frac{\xi^{2}}{4k}\right)\cdot exp\left(-\xi \cdot Z\right)\cdot \Phi \left(\frac{Z}{2\sqrt{\chi \cdot t}}-\xi \sqrt{\chi \cdot t}\right)-\right.\\ \left.-exp\left(-\xi \cdot Z\right)\cdot \Phi^{*}\left(\frac{Z}{2\sqrt{\chi \cdot t}}-\xi \sqrt{\chi \cdot t}\right)\right]d\xi , \end{array}$$

where

$J_{0}\left(\xi ,\;r\right)$ is the zero-order Bessel function;
Φ(x) is the error function; $\Phi^{*}\left(x\right)=1-\Phi (x)$.

The standard temperature field on the surface of exposure during APT can be established provided that

$$\frac{d^{2}}{4\lambda }\ll t\ll \;\frac{L^{2}}{\lambda }.$$

Then, expression (3) for the steady-state quasi-stationary mode of APT will take the following form:

(12) $$T\left(r,\;Z,\;\infty \right)=\frac{q_{0}K}{4\lambda k}\int_{0}^{\infty }J_{0}\left(\xi ,\;r\right)\cdot exp\left(-\frac{\xi^{2}}{4k}\right)\cdot exp\left(-\xi \cdot Z\right)d\xi ,$$

Based on the last expression, it is possible to estimate the maximum power density of APT at which melting of the working surface will be observed:

(13) $$q=\frac{4\sqrt{2}T\lambda }{dK}f\left(F\right),$$

where f(F) is a temperature function of the following form:

$$f\left(F\right)={\left(\pi -2\cdot \arctan{\left(8F\right)}^{-\frac{1}{2}}\right)}^{-1};$$

F is the Fourier number, $F=4\chi t/d^{2}.$

When processing with atmospheric plasma, there is a non-stationary temperature field in the material, which causes a change in the stress state. In this case, determining the stress state of the material surface during APT can be reduced to solving the following equation:

(14) $$\nabla^{2}\varphi =mT,$$

with initial and limit conditions:

$${\left.\varphi \right|}_{t=0}={\left.\frac{\partial \varphi }{\partial t}\right|}_{t=0}=0;\;\sigma_{ZZ}\left(r,0,t\right)=\sigma_{XZ}\left(r,0,t\right)=0,$$

where

φ is the thermoelastic displacement potential;
m is the material coefficient, which is calculated by the expression:

(15) $$m=\frac{\alpha \left(1+\mu \right)}{\left(1-\mu \right)};$$

α is the coefficient of thermal linear expansion;
μ is Poisson’s ratio.

During continuous APT of the surface of a sample or part material, a stationary stress field can be established. Then, the expression for radial stresses will have the following form:

(16) $$\sigma \left(r,0,\infty \right)=\frac{\alpha q_{0}KE}{4\lambda kVR_{max}}\sqrt{\pi k}\;\cdot F_{1}\left(\frac{1}{2};1;2-kr^{2}\right),$$

where

$F_{1}\left(\alpha ,\beta ,\gamma \right)$ is the form of the hypergeometric function;
E is the Young’s modulus;
$R_{max}$ is the value of the maximum protrusions of surface roughness.

Analysis of the stationary stress field during APT showed that the maximum stress values occur in the surface layer at r=0. At a certain point on the surface and time, the maximum stresses can be determined by the following expression:

(17) $$\begin{array}{c} \sigma \left(0,0,t\right)=\frac{q_{0}KG\chi }{\lambda k{fR}_{max}}\cdot \frac{\alpha \left(1+\mu \right)}{\left(1-\mu \right)}\left(\frac{\sqrt{k}\left(1+\mu \right)}{3\chi \;\sqrt{\chi t^{2}}}\cdot F_{2}\left(\frac{3}{2};2;\frac{5}{2}-\frac{1}{4k\chi t}\right)-\right.\\ \left.-\frac{k\sqrt{t}}{\left(1+4k\chi t\right)\sqrt{\pi \chi }}\cdot \frac{1-\mu }{\chi }\cdot \frac{\sqrt{k}}{\sqrt{\chi t^{2}}}\cdot F_{2}\left(\frac{1}{2};1;\frac{3}{2}-\frac{1}{4k\chi t}\right)\right), \end{array}$$

where

G is the displacement modulus of the material;
$F_{2}\left(\alpha ,\beta ,\gamma \right)$ is the form of the hypergeometric function of the second type.

Based on expressions (9), we will estimate the surface roughness of the samples after APT. As a result, the value of the maximum protrusions of surface irregularities depending on the stationary stress field during APT will take the following form:

(18) $$\begin{array}{c} R_{max}=\frac{q_{0}KG\chi }{\lambda kf\sigma \left(0,0,t\right)}\cdot \frac{\alpha \left(1+\mu \right)}{\left(1-\mu \right)}\left(\frac{\sqrt{k}\left(1+\mu \right)}{3\chi \;\sqrt{\chi t^{2}}}\cdot F_{2}\left(\frac{3}{2};2;\frac{5}{2}-\frac{1}{4k\chi t}\right)-\right.\\ \left.-\frac{k\sqrt{t}}{\left(1+4k\chi t\right)\sqrt{\pi \chi }}\cdot \frac{1-\mu }{\chi }\cdot \frac{\sqrt{k}}{\sqrt{\chi t^{2}}}\cdot F_{2}\left(\frac{1}{2};1;\frac{3}{2}-\frac{1}{4k\chi t}\right)\right). \end{array}$$

Based on model (18), optimal power and APT speed modes can be calculated for the studied sample material in order to reduce the maximum values of surface irregularity protrusions.

**Figure 2:**
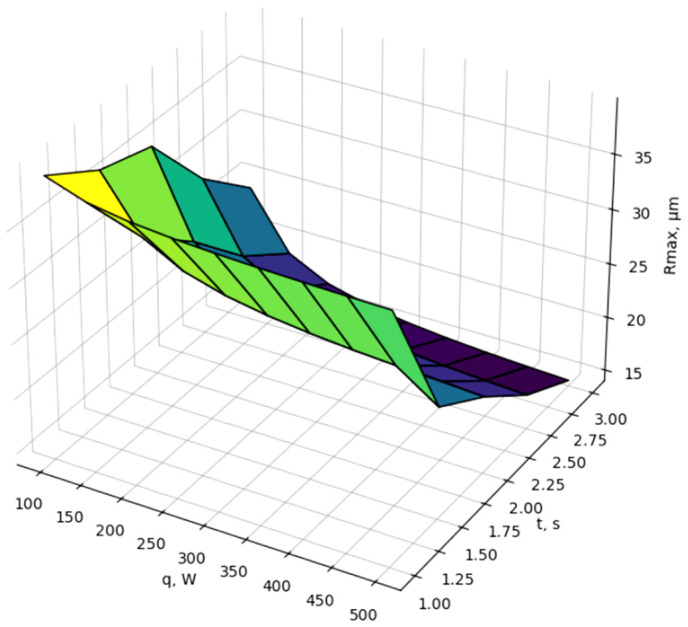
Dependence of the maximum protrusions of surface on the maximum power density of APT and the time of action of APT to the surface of the sample (carbon steel S450, measuring 10 × 10 mm) (K = 46 W/(m·K), G = 2.1 × 10^11^ Pa, χ = 0.72, λ = 46 W/(m·K), k = 0.82, f = 0.15, σ(0,0,t) = (1.8–3.4) × 10^8^ Pa; α = 1.2 × 10^−5^ K^−1^, μ = 0.29).

For the calculation example, the values of the physical and mechanical parameters for carbon steel S450 were selected. In this case, the value of the maximum protrusions of the untreated surface of the sample, measuring 10 × 10 mm, was 47 ± 4.0 μm. Based on the obtained model (18), the dependence of $R_{max}$ on the maximum power density of APT and the time of action of APT on the surface of the sample was calculated. The results are shown in Figure 2.

Figure 2 illustrates the dependence of the maximum protrusions of surface microroughness on the APT power density and the exposure time on the sample. After applying APT, the theoretical value of the height of the maximum protrusions of microroughnesses significantly decreased. An increase in the exposure time also contributes to a more uniform surface smoothing. Thus, with the APT exposure time of 1 s, the $R_{max}$ value decreased by 1.26–1.38 times compared to the initial values. With an increase in the APT exposure time to 3 s, the theoretical value of $R_{max}$ decreases to 16–23 μm. As the APT power density increases, a natural decrease in the protrusions of microroughnesses is observed. However, the optimal value of the APT power density lies in the range of 300–350 W, and the optimal ART exposure time is 2.5–3.0 s.

To confirm the theoretical prerequisites of the APT processing to reduce the maximum protrusions of surface microroughness, it is necessary to conduct experimental studies on physical samples. Then, compare the obtained experimental results with the theoretical results; validate the obtained theoretical and experimental results of the study.

### 2.3. Methods

This work investigated alloy steel, from which parts of RT components are made. The specific grade of alloy steel is not provided; however, the chemical composition of this alloy steel is shown in Table 1.

Table 1 shows that this alloy steel has a balanced composition providing strength, wear resistance and corrosion resistance. The high copper and zinc content distinguishes this alloy from standard structural steels, making it particularly suitable for parts of RT components subject to high loads and aggressive environments.

Also in Table 2 are given the mechanical properties of the studied alloy steel for a sample of size 25 × 14 × 100 mm. In this case, the ambient temperature was 20 °C.

Table 2 demonstrates that the alloy steel under study has a combination of high strength, hardness and ductility. This makes the material ideal for parts of RT components subject to significant mechanical loads, wear and impact.

The alloy steel samples were prepared as follows. The examined surface measured 25 × 14 mm, while the length of the cut alloy steel samples was 14 mm. A total of 32 alloy steel samples were prepared. The alloy steel was cut using a BP95d electro-erosion cutting machine (ZAP B.P., Kutnia, Poland), which allowed for maintaining the structural parameters of the sample material. To ensure ease of tracking the experimental outcomes, each sample was labeled with a unique identifier.

The marking was made in the form of an inscription of a number on the non-working surface of the sample using a marker. All the studied samples were prepared in the same way and processed under the same conditions.

The studied surface of the alloy steel samples was sandblasted and degreased. The roughness of the studied surface of the alloy steel samples did not change after cutting.

**Figure 3:**
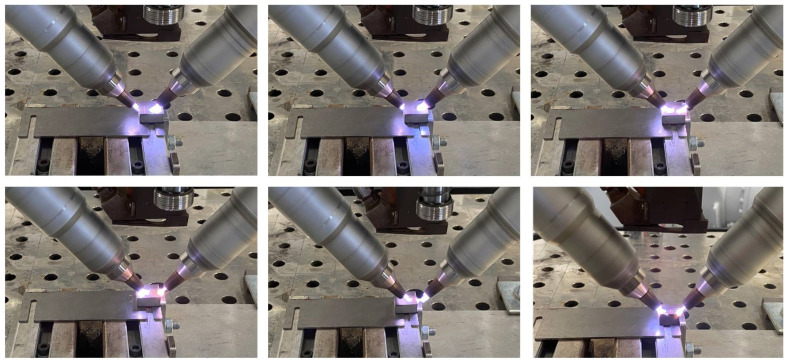
Photos depicting the procedure of atmospheric plasma surface to alloy steel samples treatment applied.

The surface of the alloy steel samples underwent APT using the TruLaser Robot 5020 system (TRUMPF GmbH + Co. KG, Ditzingen, Germany). Photographs illustrating the surface modification of the alloy steel samples during APT were taken and are shown in Figure 3.

The APT parameters for processing the surfaces of the alloy steel samples using the TruLaser Robot 5020 were defined as follows:

- Voltage: 316–318 V;
- Current: 4.1–4.3 A;
- Gas pressure: 28–29 mBar;
- Number of passes: 3;
- Speed range: 2840–2890 rpm;
- Power consumption: 342 W.

The surface roughness of the alloy steel samples was evaluated using the contact method, following the standards outlined in EN ISO 4287 [20] and EN ISO 4288 [21]. For this purpose, the Hommel-Etamic T8000RC device (HOMMEL-Etamic, Jena, Schwenningen, Germany) was employed to assess the surface geometry. A central region of 10 × 10 mm was designated on each alloy steel specimen, providing an adequate area for acquiring representative data on the surface geometry. Roughness measurements were taken perpendicular to the roughness lines.

After the roughness measurement, the result with the largest parameters $R_{a}$ and $R_{z}$ was recorded. Additional roughness parameters were measured as well; however, they are not included in this paper.

To evaluate the impact of APT on the mechanical characteristics of the alloy steel specimens, an analysis of the surface microhardness of the processed samples was conducted. A QNESS 10/60 M tester (ATM Qness GmbH, Mammelzen, Germany) was used to measure the surface microhardness of the alloy steel samples. The HV0.3 technique was employed, utilizing an indenter load of 2.94 N.

To verify the accuracy of the microhardness measurements obtained using the HV0.3 method, three separate readings were conducted at various locations on the surface of each alloy steel specimen.

Then, the average value of the surface microhardness of the samples was calculated, and a comparative analysis of the microhardness of the analyzed sample surfaces prior to and following the application of APT was carried out. An analysis of the surface microstructure of the alloy steel specimens was carried out to examine alterations in the material’s structure following APT.

The microstructure and chemical composition of the alloy steel samples were examined using a TESCAN MIRA3 scanning electron microscope (Brno, Czech Republic). The analysis employed various magnification levels to ensure a comprehensive evaluation of the microstructure. At a lower magnification of ×250, an overall assessment of the surface structure was conducted, allowing for the identification of major defects or macrostructural alterations. Higher magnification ranges (×1000 to ×10,000) enabled a more detailed examination of the microroughness, texture, and finer structural elements.

Tribological tests were carried out using a ball-on-disk configuration under dry sliding conditions, following the guidelines of ASTM G99-17 (Standard Test Method for Wear Testing with a Pin-on-Disk Apparatus) [22] and ISO 7148-2 (Plain bearings—Testing of the tribological properties of bearing materials) [23].

Two series of disk specimens were prepared: untreated samples (Base) and samples after atmospheric plasma treatment (Treated). As counterbodies, steel balls of grades 1.3529 and 1.3505 with a diameter of 6 mm were employed. Prior to testing, both disks and balls were ultrasonically cleaned in isopropanol for 5 min and dried in nitrogen to eliminate contaminants and ensure consistent initial conditions.

The test parameters were defined as follows:

Normal load (*P*): 10 N;
Sliding speed (*v*): 0.20 m/;
Total sliding distance (*L* = 2*πR·N*): 300 m;
Track radius (*R*): Fixed at 15 mm (corresponding to *N* ≈ 318 revolutions for *L*);
Ambient conditions: Temperature 22–25 °C, relative humidity 30–35%.

During the tests, the friction coefficient μ(t) was recorded continuously at a sampling frequency of 10 Hz. The values were averaged over the steady-state region of sliding. The stability of friction was quantified using the coefficient of variation (CV) of μ in the steady-state regime.

Wear of the disk specimens was evaluated by 3D profilometry of the wear track (confocal/white light profilometer). Cross-sectional profiles were integrated across the wear track to determine the volumetric loss ΔV. The specific wear rate was calculated according to

*W_s_* = Δ*V*/(*P·L*), [mm^3^/(N·m)]. (19)

Counterbody wear was determined by measuring the diameter of the wear scar under an optical microscope and calculating the lost spherical segment volume.

Each experimental condition (state × counterbody) was tested in triplicate (n = 3) using a fresh ball and a new track for each run. Statistical analysis included two-tailed *t*-tests (*α* = 0.05) for pairwise comparisons and one-way ANOVA with Tukey post hoc tests for multigroup comparisons. Outlier trajectories were excluded only if instrument malfunction or misalignment was detected. Repeatability was verified through overlapping confidence intervals of *μ* and *W_s_* values for independent replicates.

High-temperature tribological tests were carried out using the same ball-on-disk configuration as in the room-temperature experiments, with additional controlled heating of the disk specimen. Heating was performed using an electric resistance heating stage integrated beneath the specimen holder. The temperature in the contact region was monitored using a K-type thermocouple positioned near the wear track area and calibrated prior to testing using a reference digital temperature controller with an accuracy of ±2 °C. The experiments were conducted at temperatures of 25, 100, 200, 300, and 370 °C. Before each test, the specimen was heated to the target temperature and maintained for 10 min under isothermal conditions to ensure thermal stabilization of the contact zone. During testing, the temperature deviation did not exceed ±5 °C. The experimental procedure included ultrasonic cleaning of the disk and counterbody in isopropanol, mounting of the specimen on the heated stage, heating to the required temperature, thermal stabilization, tribological testing under dry sliding conditions, and subsequent cooling before wear track characterization. The testing parameters remained identical to those used at room temperature: normal load 10 N, sliding speed 0.20 m/s, sliding distance 300 m, and track radius 15 mm.

## 3. Results and Discussion

### 3.1. Surface Roughness of Alloy Steel Samples

**Figure 4:**
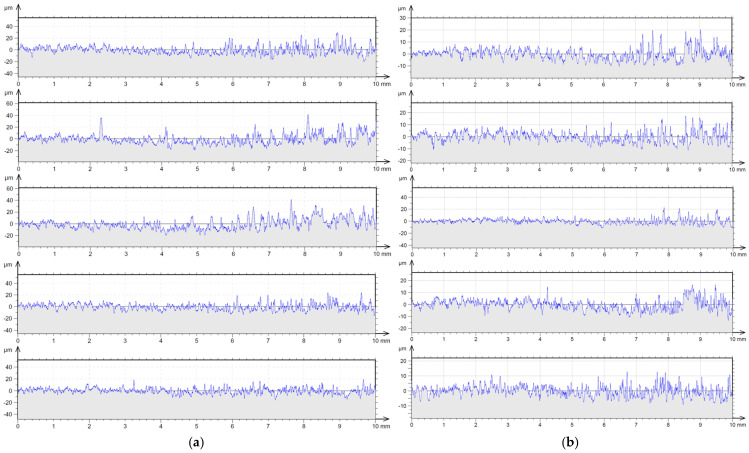
Some results of roughness measurements: (**a**) of basic prepared samples; (**b**) after APT.

The surface roughness of the prepared specimens, both before and after APT, was assessed using the procedure. For clarity, Figure 4 presents two sets of results illustrating the roughness measurements taken before and after APT application.

The obtained measurements and the data presented (Figure 4a) indicate that the roughness parameter $R_{a}$ for the untreated sample surfaces ranged between 3.25 and 3.90 μm. Similarly, the roughness parameter $R_{z}$ for the initial samples was observed to be within the range of 20.80 to 23.20 μm.

The roughness measurements conducted, along with the data illustrated in Figure 4b, indicate that the roughness parameter $R_{a}$ for the sample surfaces treated with atmospheric plasma ranged between 2.09 and 2.38 μm. This demonstrates a reduction in the $R_{a}$ parameter by 35.7–38.9% when compared to the untreated baseline samples.

After APT, the roughness parameter $R_{z}$ for the surface of the prepared samples ranged between 13.10 and 15.40 μm. This indicates a reduction of 36.4% to 37.0% in the $R_{z}$ parameter compared to the untreated base samples.

Figure 4 confirms that APT significantly reduces the surface roughness, making it a promising method for industrial applications.

The theoretical parameter $R_{max}$ (Equation (18)) used in the proposed model characterizes the maximum height of surface asperities and is physically closest to the experimentally measured roughness parameter $R_{z}$, which represents the average maximum peak-to-valley height within the sampling length according to EN ISO 4287 [20]. In contrast, the parameter $R_{a}$ characterizes the arithmetic average deviation in the roughness profile and therefore reflects the general surface quality rather than the extreme asperity geometry. For this reason, the experimental parameter $R_{z}$ was considered the most appropriate parameter for indirect validation of the proposed model.

**Figure 5:**
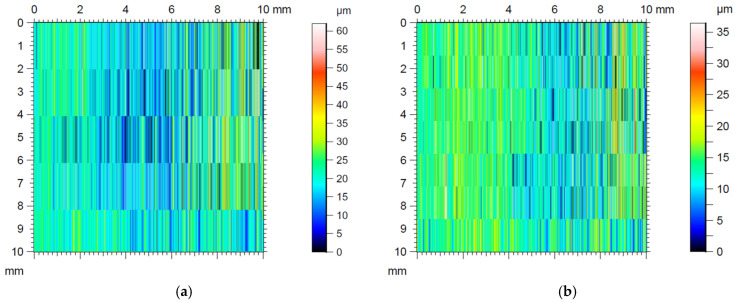
Surface roughness model of alloy steel samples: (**a**) of basic prepared samples; (**b**) after APT.

As part of the study, measurements of the surface roughness of the alloy steel samples were carried out both prior to and following APT. Based on the findings, a generalized model of the surface roughness was developed, with the outcome presented in Figure 5.

The models shown in Figure 5 exhibit comparable roughness topologies. Nevertheless, the heights of protrusions and depths of depressions on the surfaces of the alloy steel samples treated with APT (Figure 5b) are reduced by a factor of 1.5–1.7 compared to the surface roughness of the untreated samples. This demonstrates the potential of APT as an effective method for minimizing surface roughness in alloy steel samples.

Comparison between the theoretical predictions and experimental measurements demonstrates good agreement. The mathematical model (18) predicts a decrease in the characteristic asperity height by approximately 1.3–1.7 times depending on the atmospheric plasma treatment conditions, while the experimentally measured $R_{z}$ values decreased by approximately 36–37% after APT. Thus, both the theoretical and experimental results confirm the same tendency of a significant reduction in the surface asperity height after plasma treatment.

### 3.2. Microhardness Study Results

The examination of the microhardness of the alloy steel samples, conducted both prior to and following APT, revealed that the microhardness values ranged between 527 and 637 HV.

The results of the study of the microhardness measurements of the alloy steel samples after APT are given in Table 3.

From Table 3, we see that the average value of the microhardness of the alloy steel samples after APT differs by less than 1.34%.

The results of the microhardness measurements of the base alloy steel samples are similar to the microhardness values of the processed samples. The average microhardness values of the base alloy steel samples were in the range of 579.33–580.67 HV. The standard deviation varies in the range of 27.16–50.13 HV, indicating a small variability in the results, confirming the reliability of the method. Compared with the untreated samples, no significant changes in microhardness are observed, proving that APT does not degrade the strength characteristics of the material.

The microhardness measurement results for the alloy steel samples subjected to APT indicate that the mechanical properties of the material remain unaffected. This demonstrates the potential of atmospheric plasma for reducing the surface roughness of alloy steel samples.

The obtained data show that APT does not reduce the alloy hardness. This is especially important, since most thermal or mechanical treatments can weaken the material.

APT treatment reduces the surface microroughness, but does not change the internal structure. This is confirmed by the stable microhardness values.

Table 3 shows that APT can be used to improve the surface characteristics without compromising the mechanical properties. This opens up prospects for the application of this technology in railway engineering.

### 3.3. The Findings from the Analysis of the Microstructure of the Samples Following APT

**Figure 6:**
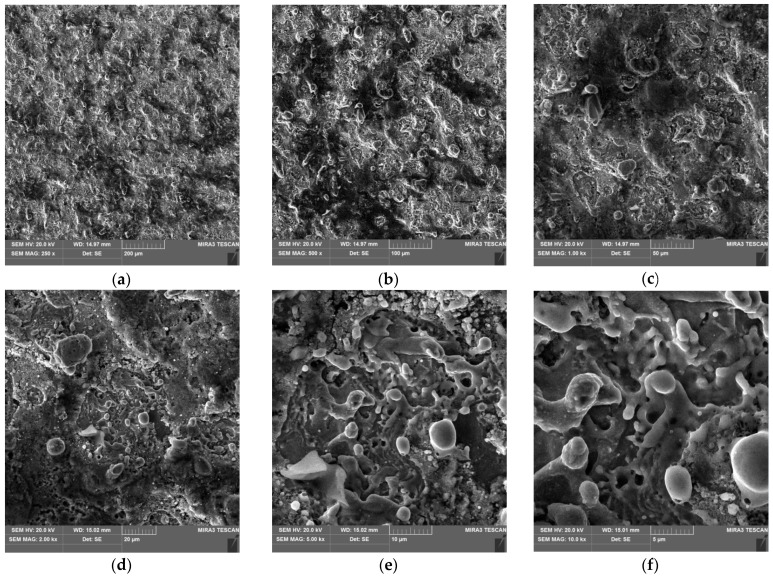
Microstructure of alloy steel samples after APT: (**a**) ×250; (**b**) ×500; (**c**) ×1000; (**d**) ×2000; (**e**) ×5000; (**f**) ×10,000.

Figure 6 presents the microstructure of the alloy steel samples following the application of APT.

The electron microscopy images of the microstructure of the alloy steel samples subjected to APT (Figure 6) reveal a decrease in surface irregularities and a more uniform texture, aligning with the findings from the roughness measurements.

As shown in Figure 6, melted formations can be observed on the surface of the alloy steel samples following APT, providing evidence of reduced roughness, as demonstrated in the earlier results. The interaction of atmospheric plasma with surface irregularities involves melting the protruding elements, which results in a decrease in roughness values. Notably, the material’s structure in the deeper layers of the alloy steel samples remains intact after atmospheric plasma exposure, indicating a minimal impact of the plasma on the material and preserving its fundamental properties.

In addition to the observed smoothing effect, the obtained microstructural features suggest that atmospheric plasma treatment may also influence the near-surface physicochemical state of the alloy steel. The localized thermal cycling associated with plasma exposure can promote the partial redistribution of residual stresses and the formation of thin re-solidified surface layers. Furthermore, the interaction of the plasma jet with the atmospheric environment may contribute to the formation of ultrathin oxide-containing films and the modification of surface energy, which can additionally affect the friction stability and wear behavior. Although the present SEM observations confirm localized melting and re-solidification phenomena, more detailed characterization techniques such as XPS, TEM, EBSD, or XRD residual stress analysis would be required to fully identify nanoscale phase transformations, oxide chemistry, and stress redistribution mechanisms. Therefore, these effects should be considered as probable contributing factors to the improved tribological performance observed after APT.

Overall, the following conclusions can be made based on Figure 6:

- Smoothing of the surface after APT. When compared to the untreated surface, it is evident that the height of the peaks and the depth of the valleys of the microroughnesses have decreased significantly.
- This confirms the results of the roughness measurements (Figure 4 and Figure 5), where a decrease in the $R_{a}$ and $R_{z}$ parameters was observed.
- Formation of smooth transitions in the microstructure. At low magnifications (×250–×1000), it can be seen that the surface has become more uniform. High magnifications (×2000–×10,000) show that melted and re-solidified areas are present, which indicates the thermal effect of APT.
- Preservation of the basic structure in the depth of the material. Despite the melting of the surface microroughnesses, the deep layers of the material have not undergone significant changes. This confirms that APT does not have a destructive effect on the main volume of the alloy, preserving its mechanical properties.
- Confirmation of the effectiveness of APT. Metallographic changes are limited to the surface layer only, which proves the high accuracy and minimal invasiveness of the method. The microstructure remains stable, which confirms the preservation of the strength properties of the material.

### 3.4. Chemical Composition of the Surface of Alloy Steel Samples After APT

**Figure 7:**
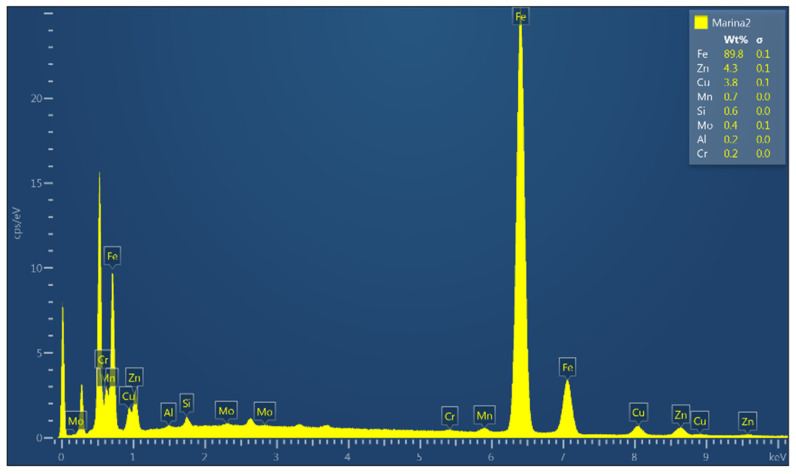
Chemical composition of the surface of alloy steel samples after APT.

The result of determining the chemical composition of the alloy steel samples after APT is shown in Figure 7.

From Figure 7, it can be observed that the chemical composition of the alloy steel surface after APT remains nearly consistent with the initial values provided in Table 1. The variation in the percentage composition between the untreated base samples and alloy steel samples subjected to atmospheric plasma does not exceed 0.2%. These findings demonstrate that APT does not alter the chemical composition of alloy steel. This aspect is crucial and should be considered in future studies, as it highlights that APT preserves the material’s chemical integrity. In contrast, most existing methods designed to enhance the mechanical properties of surfaces typically induce changes in the chemical composition of the material’s surface.

It should be noted, however, that the EDS analysis performed in this study characterizes the average elemental composition within the analyzed interaction volume and may not detect ultrathin oxide films or subtle changes in the chemical states of surface atoms formed during atmospheric plasma exposure. Therefore, although no significant variations in bulk elemental composition were observed, the formation of nanoscale oxide-containing layers or surface activation effects cannot be completely excluded. Such modifications may contribute to the improved friction stability and reduced wear observed after APT. More surface-sensitive techniques, particularly X-ray photoelectron spectroscopy, would be required to clarify the chemical bonding states and near-surface oxide chemistry induced by plasma treatment.

Figure 7 confirms that after APT, the percentage of these elements remains unchanged, indicating no burn-off or diffusion of alloying elements.

Many traditional surface treatment methods (grinding, laser or heat treatment) can change the surface chemistry through oxidation, evaporation of alloying elements or diffusion.

However, Figure 7 demonstrates that APT does not result in the loss of key alloying elements, making this method preferable for improving the performance of railway components.

APT does not change the chemical composition of the alloy, which is an important advantage of the method, since it does not affect the strength of the material.

This confirms the effectiveness of the treatment, making APT a better alternative to mechanical or chemical treatment.

This result, along with the data on roughness reduction (Figure 4 and Figure 5), confirms that APT improves the surface without changing its composition.

### 3.5. Wear Test Results

**Figure 8:**
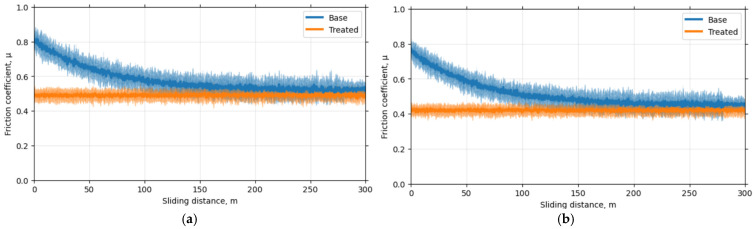
Results of the obtained friction coefficient during testing of a steel plate with a counterbody made of steel 1.3529 (**a**) and with a counterbody made of steel 1.3505 (**b**).

Ball-on-disk tests (dry friction, 25 °C) with a load of 10 N, a speed of 0.20 m/s and a total distance of 300 m showed a decrease in the friction coefficient and wear of the steel plate after surface treatment for both 1.3529 and 1.3505 steel counterbodies. The results of the obtained friction coefficient during the tests are shown in Figure 8.

Figure 8 presents the friction coefficient μ as a function of the sliding distance during ball-on-disk tests for a steel plate before (Base) and after APT (Treated), using counterbodies made of steels 1.3529 (Figure 8a) and 1.3505 (Figure 8b).

For the untreated surface, a pronounced running-in stage is observed up to ~250–260 m, during which μ gradually decreases from elevated initial values to a steady-state level. This behavior indicates unstable contact conditions and the active smoothing of surface asperities through wear processes.

In contrast, for the treated surface, the friction coefficient immediately reaches a plateau and remains stable throughout the test. The absence of a long running-in stage reflects the more uniform microtopography and enhanced contact properties of the plasma-modified surface.

The observed reduction in the friction coefficient after APT is consistent with the established relationship between the surface morphology, interfacial contact conditions, and energy dissipation mechanisms during sliding. In previous studies [24,25,26], it was shown that friction is strongly governed by asperity-scale interactions, adhesion, local thermal dissipation, and the evolution of surface topography during contact. In particular, smoother and more uniform surfaces with reduced asperity heights exhibit lower friction coefficients and improved stability of sliding behavior. It was also demonstrated that multiscale surface roughness affects thermal dissipation and contact-state evolution, thereby influencing the friction dynamics and wear processes. The present results are in good agreement with these findings, since atmospheric plasma treatment reduced the asperity height, shortened the running-in stage, stabilized the friction regime, and decreased the wear intensity. Therefore, the improved tribological performance after APT can be associated not only with macroscopic surface smoothing, but also with more stable asperity-scale interactions and reduced interfacial energy dissipation during sliding contact.

The improved tribological behavior observed after APT is likely associated not only with the reduction in the surface roughness, but also with additional physicochemical modifications of the near-surface layer. Localized plasma-induced heating and rapid re-solidification may contribute to the redistribution of residual stresses and stabilization of the contact layer during sliding. In addition, the interaction between the plasma jet and atmospheric oxygen can promote the formation of thin oxide-containing surface films, which may act as protective tribolayers and reduce adhesive interactions. Surface activation effects and possible nanoscale structural rearrangements within the re-solidified layer may also contribute to the observed reduction in friction coefficient fluctuations and wear intensity. Although these mechanisms were not directly characterized in the present work, the obtained tribological results are consistent with such combined effects. Further studies using surface-sensitive techniques such as XPS, TEM, EBSD, and XRD residual stress analysis are planned to clarify the contribution of these factors in greater detail.

Numerically, the steady-state values are:

For the pair with steel 1.3529: μ decreased from 0.52 ± 0.04 (Base) to 0.46 ± 0.03 (Treated), corresponding to a reduction of 10.4–12.5%.

For the pair with steel 1.3505: μ decreased from 0.45 ± 0.03 (Base) to 0.40 ± 0.03 (Treated), corresponding to a reduction of 10.4–11.9%.

In addition to lowering the absolute values of μ, plasma treatment also reduced the amplitude of fluctuations in the friction coefficient, which indicates a more stable tribological behavior.

**Figure 9:**
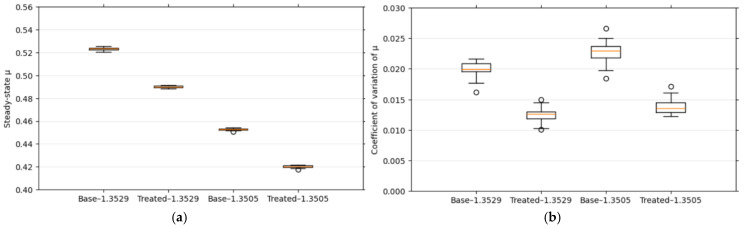
Results of calculations of the median coefficient of friction (**a**) and results of calculations of the coefficient of variation of the coefficient of friction in a stationary section (**b**).

For a better explanation of Figure 8, clarifying mathematical calculations were performed. In this case, the results of calculating the value of the median friction coefficient μ are shown in Figure 9a, and the results of calculating the value of the coefficient of variation of the friction coefficient in the stationary section are shown in Figure 9b.

The results of calculations of the median coefficient of friction (Figure 9a) indicate the following:

- For a pair with a counterbody made of steel 1.3529, the median μ on the machined surface of the steel plate is lower by ~6% than that of the untreated one;
- For a pair with a counterbody made of steel 1.3505, the median μ on the machined surface of the steel plate is lower by ~7% than that of the untreated one.

The range of variation in the median coefficient of friction on the machined surface of the steel plate is smaller in both cases, which indicates a narrower distribution of μ values and better test reproducibility.

A lower value of the coefficient of variation of the friction coefficient is consistent with the fact that on the treated surface of the steel plate after entering the friction mode, there is no long-term running-in or no running-in at all, and the amplitude of fluctuations μ is lower, which means that the contact operates in a more stable friction mechanism.

**Figure 10:**
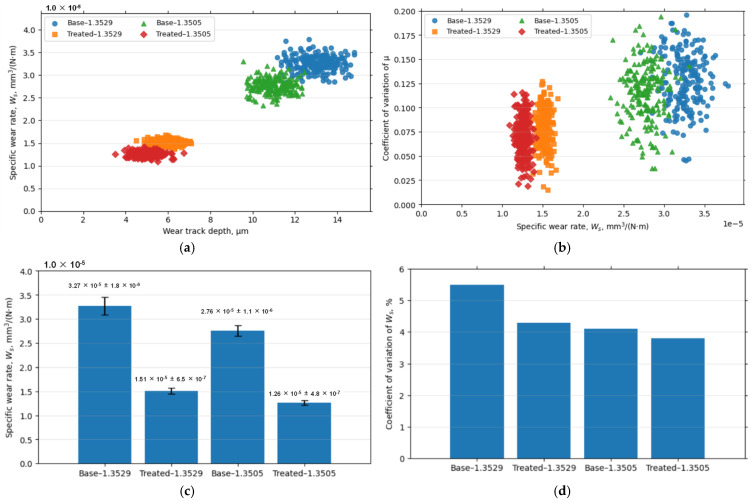
Correlation of wear track depth measurements and calculation of the specific wear coefficient *W*_*s*_ (**a**); correlation of the coefficient of variation of the friction coefficient on a stationary section and the value of the specific wear coefficient *W*_*s*_ (**b**); results of calculations of the average value of the specific wear coefficient *W*_*s*_ (**c**) and the coefficient of variation (**d**).

The correlation of measurements and calculation of the specific wear coefficient *W*_*s*_, wear track depth and the coefficient of variation of the friction coefficient on a stationary section are shown in Figure 10a,b. The results of the calculations of the average value of the specific wear coefficient *W*_*s*_ and the coefficient of variation are shown in Figure 10c,d.

Figure 10 illustrates the relationship between the wear track depth, the specific wear coefficient (*W*_*s*_), and the stability of the friction process for untreated (Base) and plasma-treated (Treated) steel surfaces. The untreated samples exhibit deeper wear tracks, higher (*W*_*s*_) values, and larger fluctuations in the friction coefficient, indicating unstable abrasive–adhesive wear accompanied by intensive tribo-oxidation. In contrast, the plasma-treated surfaces show shallower wear tracks, more than twofold lower (W_s_) values, and significantly reduced variation in the friction coefficient, reflecting a more stable tribological regime.

For the pair with steel 1.3529, *W*_*s*_ decreases from 3.27 × 10^−5^ to 1.51 × 10^−5^ mm^3^/(N·m), while for the pair with steel 1.3505, it decreases from 2.76 × 10^−5^ to 1.26 × 10^−5^ mm^3^/(N·m). This reduction confirms that surface modification significantly reduces the intensity of wear, making the treated surfaces more resistant to material removal under sliding contact. In practical terms, this translates to the extended service life of the component and reduced maintenance requirements for railway systems.

Overall, the results indicate that atmospheric plasma treatment promotes a transition from abrasive–adhesive wear with unstable contact behavior to a milder abrasive wear mechanism with reduced material transfer and stabilized asperity-scale interactions.

**Figure 11:**
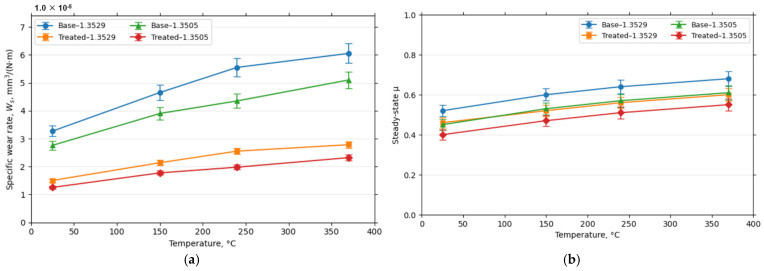
Dependences of the specific wear coefficient *Ws* (**a**) and the median friction coefficient (**b**) on temperature.

This indicates the stability of the friction process in the case of tests with the treated surface of the steel plate and both pairs of counterbodies 1.3529 and 1.3505. At elevated temperatures (25–370 °C), the treated surface of the steel plate has lower values of *W*_*s*_ and μ (Figure 11). At the same time, the values of *W*_*s*_ and μ increase with the temperature for both series, but with a smaller gradient for the treated surface of the steel plate (Figure 11a,b).

Figure 11a shows the dependence of the specific wear coefficient on the temperature in the range of 25–370 °C. For untreated (Base) surfaces, *W*_*s*_ grows rapidly with the increasing temperature, indicating accelerated wear due to softening of the near-surface layer, oxidation, and activation of adhesive interactions. In contrast, treated (Treated) surfaces demonstrate consistently lower *W*_*s*_ values across the entire temperature range, with a slower rate of growth. This suggests that APT stabilizes the surface, creating a more resistant layer that better withstands thermal stresses. The reduced sensitivity of *W*_*s*_ to the temperature rise means that treated surfaces maintain their wear resistance under more severe operating conditions, which is crucial for railway components exposed to frictional heating.

Figure 11b presents the dependence of the median friction coefficient on the temperature. As with *W*_*s*_, both series show an upward trend, reflecting the thermally activated nature of friction and wear processes. However, the plasma-treated samples maintain lower *μ* values at every tested temperature. The difference becomes particularly important at elevated temperatures, where untreated surfaces demonstrate sharp increases in μ due to the intensification of tribo-oxidation and adhesive junction formation, while treated surfaces exhibit more moderate growth. This indicates that APT not only reduces the absolute friction level but also suppresses unstable fluctuations that typically occur at high temperatures.

Overall, Figure 11 confirms the thermal stability of the tribological improvements provided by plasma treatment. The treated surfaces maintain lower friction and wear coefficients even as the temperature increases, which points to a shift in the dominant wear mechanisms—from destructive adhesive–oxidative interactions on untreated samples to a more stable abrasive mechanism with limited oxidation for treated ones. For practical applications, this means that components treated with atmospheric plasma will perform more reliably under high thermal loads, reducing the risk of overheating, accelerated wear, and premature failure.

**Figure 12:**
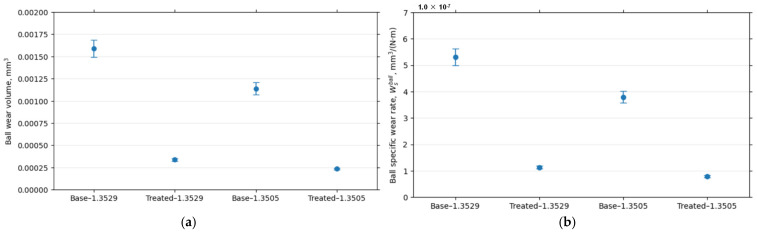
Counterbody volume wear dependencies (**a**); calculation results of the average value of the specific wear coefficient of the counterbody (**b**).

In addition, wear measurements were taken by the volume of counterbodies made of 1.3529 and 1.3505 steel during tests with the treated and base surface of the steel plate (Figure 12a). The average value of the specific wear coefficient of the counterbodies was also calculated (Figure 12b).

Figure 12 illustrates the influence of atmospheric plasma treatment on counterbody wear during sliding contact. Untreated (Base) surfaces produce significantly higher wear of the counterbodies for both steels 1.3529 and 1.3505, resulting in larger wear scars and higher material loss. This behavior is associated with unstable abrasive–adhesive interactions caused by rough surface asperities and intensive material transfer during friction.

In contrast, plasma-treated (Treated) surfaces substantially reduce counterbody wear due to smoother surface morphology and stabilization of the friction regime. The calculated specific wear coefficient of the counterbodies also decreases after APT, confirming that plasma treatment reduces not only the wear of the steel plate itself but also the degradation of the interacting body.

Overall, the results demonstrate that atmospheric plasma treatment promotes a more balanced tribological regime characterized by lower friction fluctuations, reduced material transfer, and stabilization of asperity-scale contact interactions. This transition from severe abrasive–adhesive wear to a milder abrasive mechanism is especially important for railway tribosystems operating under long-term cyclic loading conditions.

### 3.6. Comparison with Previous Studies

To highlight the novelty and practical relevance of the present research, Table 4 summarizes and compares the main findings of earlier studies with the results obtained here.

The comparison clearly demonstrates that, while earlier studies established the potential of plasma-based techniques for improving the surface finish and in some cases enhancing the mechanical behavior, they were limited either to different materials (silica, stainless steel, additively manufactured alloys) or to processes carried out under controlled or vacuum environments. In contrast, the present work provides the first systematic evaluation of the atmospheric plasma treatment of alloy steels used in railway transport, showing that APT not only reduces surface roughness by 35–37% but also leads to a 6–7% reduction in the steady-state friction coefficient, a more than twofold reduction in the specific wear coefficient, and significant stabilization of the tribological regime. Importantly, these improvements were achieved without altering the microhardness or chemical composition of the steel, confirming the eco-friendly and non-destructive nature of the method. Thus, the study fills a critical gap in the literature and positions APT as a practical, cost-effective, and scalable technology for enhancing the performance and durability of railway components.

## 4. Conclusions

This study demonstrated that atmospheric plasma treatment effectively improves the surface and tribological properties of alloy steel used in railway transport components. APT reduced the surface roughness parameters by approximately 35–37%, resulting in smoother and more homogeneous surface morphology caused by localized melting and re-solidification of surface asperities.

Tribological tests showed that plasma-treated surfaces exhibit lower friction coefficients, reduced wear intensity, improved stability of the friction regime, and significantly lower counterbody wear compared with untreated samples. The specific wear coefficient decreased more than twofold after treatment, while the coefficient of variation of the friction coefficient was substantially reduced, indicating the stabilization of asperity-scale contact interactions and transition toward a milder abrasive wear mechanism.

Microhardness measurements and chemical composition analysis confirmed that atmospheric plasma treatment does not significantly alter the bulk mechanical and chemical properties of the investigated alloy steel. This demonstrates the localized and non-destructive nature of the treatment.

The proposed mathematical model adequately describes the reduction in the surface asperity height during atmospheric plasma treatment and shows good agreement with the experimental roughness measurements.

Overall, the obtained results confirm that atmospheric plasma treatment is a promising and scalable surface engineering method for railway tribosystems, capable of improving wear resistance and friction stability while preserving the integrity of the base material. Future work should focus on advanced surface-sensitive characterization and long-term operational testing under real railway service conditions.

Future research should focus on a more detailed investigation of the physicochemical and tribological mechanisms associated with the atmospheric plasma treatment of railway alloy steels. In particular, advanced surface-sensitive characterization techniques such as X-ray photoelectron spectroscopy (XPS), Auger electron spectroscopy (AES), transmission electron microscopy (TEM), electron backscatter diffraction (EBSD), and X-ray residual stress analysis should be employed to clarify possible nanoscale phase transformations, oxide-layer formation, stress redistribution, and tribofilm evolution induced by plasma exposure. Additional studies involving SEM/EDS analysis of wear tracks and wear debris after tribological testing are also necessary to provide direct confirmation of the proposed transition from abrasive–adhesive wear to a more stable mild abrasive wear mechanism. Furthermore, future work should include long-term durability tests under real railway operating conditions and the optimization of atmospheric plasma treatment parameters for different alloy systems and component geometries.

## Figures and Tables

**Table 1:** Chemical composition of alloy steel material.

Element	Al	Si	Cr	Mn	Cu	Zn	Mo	C	Fe
%	0.20	0.60	0.20	0.70	3.70	4.20	0.30	0.20	89.80

**Table 2:** Mechanical properties of the studied alloy steel material for a sample of size of 25 × 14 × 100 mm.

Hardness, HRC	Yield Strength, σ_0.2_, MPa	Temporary Tear Strength, σ_b_, MPa	Elongation at Break, δ, %	Relative Constriction, ψ, %	Impact Strength KCU, kJ/m^2^
51.9	981	1436	24	35	103

**Table 3:** Results of measuring the microhardness of alloy steel samples after APT, HV.

Measuring Point No.	Sample No.											
	1, 16, 24	2, 12, 28	3, 6, 15	4, 14, 25	5, 13, 32	7, 18, 21	8, 9, 31	10, 30	11, 20, 29	22, 23	17, 26	19, 27
1	635	626	619	611	548	629	571	635	633	548	562	637
2	541	536	595	565	624	539	547	545	543	632	557	541
3	563	576	527	563	567	574	620	561	565	561	622	564
Mean value	$\frac{579.67^{\;*}}{580.00}$	$\frac{579.33}{579.00}$	$\frac{580.33}{580.00}$	$\frac{579.67}{580.00}$	$\frac{579.67}{580.00}$	$\frac{580.67}{580.67}$	$\frac{579.33}{579.00}$	$\frac{580.33}{580.00}$	$\frac{580.33}{580.00}$	$\frac{580.33}{580.00}$	$\frac{580.33}{580.00}$	$\frac{580.67}{580.67}$
Mean square deviation	$\frac{49.17^{\;*}}{48.77}$	$\frac{45.10}{45.57}$	$\frac{47.73}{47.57}$	$\frac{27.16}{26.89}$	$\frac{39.56}{40.11}$	$\frac{45.37}{44.38}$	$\frac{37.21}{37.64}$	$\frac{48.02}{48.38}$	$\frac{46.92}{47.32}$	$\frac{45.22}{44.64}$	$\frac{36.18}{36.50}$	$\frac{50.13}{50.12}$

* The numerator shows the values after APT treatment; the denominator shows the values before treatment.

**Table 4:** Comparison of obtained results with previous studies on plasma-based surface treatments.

Author(s) & Year	Material	Method	Key Findings in Earlier Studies	Comparison with Present Work (APT on Railway Steels)
Lyu et al. (2025) [15]	Fused silica	Laser-enhanced plasma polishing	Achieved atomic/near-atomic smoothness; focused on optical performance.	Similar smoothing principle observed; our work demonstrates tribological benefits (friction & wear reduction) on steels, not just topography.
Inoue & Furushima (2024) [16]	Metals for microforming	Modeling of surface roughening	Showed strong effect of roughness evolution on forming limit curves.	Confirms importance of surface topography; our study shows APT directly improves tribological behavior under sliding wear.
Fischer & Stöbener (2019) [17]	Rolled sheet metals	In-process roughness inspection	Enabled real-time control of surface roughness in rolling.	Diagnostic approach; in contrast, APT provides corrective modification, reducing roughness by 35–37%.
Yamaguchi et al. (2017) [18]	Additively manufactured parts	Magnetic field-assisted finishing	Reduced surface roughness and residual stresses; improved fatigue behavior.	Complex hybrid setup required; APT achieves comparable surface improvements in steels with simpler, scalable technology.
Deng et al. (2022) [19]	Stainless steel (AISI 304)	Plasma beam polishing	Significant roughness reduction with optimized parameters.	Our results confirm >35% roughness reduction and extend analysis to tribological properties, showing >2× lower wear coefficient.
Sharma & Kumar (2020) [13]	Structural steels	Plasma arc cutting	Optimized dross formation, improved cut edge surface quality.	Their focus was on cutting; our study applies plasma for controlled surface modification under atmospheric conditions.
Das & Chakraborty (2023) [14]	Structural steels	Plasma arc cutting optimization	Developed multi-criteria parametric optimization for better surface finish.	Supports the sensitivity of plasma processes to parameters; our model links APT parameters directly to asperity height reduction.

## Data Availability

Data are contained within the article.
